# Kidney Stones of Type I vs. Type II Diabetic Patients: Are There Any Differences?

**DOI:** 10.3390/jcm13206110

**Published:** 2024-10-14

**Authors:** Cătălin Pricop, Marius Ivănuță, Mihaela Nikolic, Dragoş Puia

**Affiliations:** 1 “Grigore T. Popa”, Faculty of Medicine, University of Medicine and Pharmacy, 700115 Iasi, Romania; catalin.pricop@umfiasi.ro (C.P.); dragos-puia@umfiasi.ro (D.P.); 2 Department of Urology, “Dr. C.I. Parhon” Clinical Hospital, 700503 Iasi, Romania; 3 Center for Morphological and Spectroscopic Analysis of Urinary Stones “Michel Daudon”, 700503 Iasi, Romania; bomibo2425@yahoo.com; 4 “Ion Ionescu de la Brad” Iasi University of Life Sciences, 700503 Iasi, Romania

**Keywords:** kidney stones, diabetes, spectroscopy, lithiasis recurrence, stone composition

## Abstract

**Background**: This study highlighted the differences between the biochemical compositions of urinary stones from patients with type 1 diabetes versus those with type 2 diabetes. **Materials and Methods**: This study included patients diagnosed with kidney stones and diabetes who were referred to the Urological Clinic of the Dr. C. I. Parhon Hospital in Iasi from April 2017 to April 2024. We analyzed the spectroscopic stone composition from 128 lithiasis patients treated in our Clinic. In the current study, the distribution of the biochemical composition of stones varied significantly between diabetic patients with type 2 diabetes, who formed primarily mixed uric acid stones, and diabetic patients with type 1 diabetes, who mainly developed pure uric acid stones (*p* < 0.001). Patients with uric acid stones had significantly higher mean creatinine values than the other stone types (*p* < 0.001). Urinary pH levels were abnormal for all biochemical subtypes of stones, indicating acidic urine. However, patients with uric acid stones had lower pH values than the group average. From the Kaplan–Mayer analysis, patients with pure uric acid stones had a shorter time to stone recurrence compared to patients with other biochemical types identified. **Conclusions**: These findings, which highlight the prevalence of pure uric acid stones in patients with type 1 diabetes and the impact of this on the strategy for dissolving pure stones, represent a significant advancement in understanding urinary lithiasis in diabetic patients.

## 1. Introduction

Over the last few decades, there has been a rise in both the incidence and frequency of urolithiasis in most developed countries, along with an increase in the prevalence of metabolic syndrome, obesity, and diabetes [1]. These epidemiological changes are associated with significant dietary and lifestyle modifications [2]. Approximately 60 million individuals in Europe have diabetes, which accounts for approximately 10.3% of men and 9.6% of women aged 25 years and older. The occurrence of diabetes is on the rise across all age groups in Europe, primarily as a result of the growing rates of overweight and obesity, poor dietary habits, and lack of physical activity. The World Health Organization (WHO) predicts that the number of deaths caused by diabetes will increase twofold from 2005 to 2030 [3,4]. On the other hand, renal stones increase the patient’s susceptibility to chronic kidney disease (CKD), end-stage renal disease (ESRD), hypertension, and metabolic bone disease (MBD) [5]. Although urinary lithiasis is not commonly recognized as a classic complication of diabetes, recent studies on the association between diabetes and metabolic syndrome suggest that these widely encountered conditions are associated with the development and recurrence of urinary lithiasis. Meydan et al. [6] found that the prevalence of kidney stone disease among 321 diabetes patients was 21%, but among 115 age-matched nondiabetic control subjects, the prevalence was 8%. In another epidemiological analysis, Daudon et al. found a significantly higher percentage of type 2 diabetes patients among those who formed uric acid (UA) stones (28.2%) compared to those who formed calcium stones (8.2%) [7]. Lieske et al. conducted a study in which they highlighted that the prevalence of diabetes was significantly higher in UA stone formers (40%) compared to calcium stone formers (9%), with four out of ten UA stone formers being diabetic [8]. Few studies explore the differences in the spectroscopic composition of kidney stones between patients with type 1 diabetes and patients with type 2 diabetes. Most research focuses on the overall incidence of kidney stones among diabetic patients without making a detailed differentiation between types of diabetes. Thus, there is a lack of comparable data on the biochemical composition of stones in these two categories of patients, which limits our understanding of the specific influence of each type of diabetes on the formation of kidney stones. In our research, we analyzed the spectroscopic characteristics of urinary calculi from patients with type 1 diabetes versus patients with type 2 diabetes. We also examined the risk factors associated with patients’ likelihood of recurrence.

## 2. Materials and Methods

This study employed a retrospective observational design to evaluate risk factors, clinical patterns, and stone composition in patients with concomitant diabetes mellitus and urolithiasis. All patients were referred to the Urological Clinic of Dr. C. I. Parhon Hospital in Iasi, Romania, from April 2017 to April 2024. The objective was to identify main predictors of kidney stone recurrence and evaluate the influence of diabetes on lithiasis.

Inclusion criteria were adult patient (aged 18 years and above), confirmed diagnosis of urolithiasis, with either spontaneous passage or postinterventional stone extraction, concurrent diagnosis of diabetes mellitus (either type 1 or type 2), patients who provided informed consent for participation in the study and use their medical data for research purposes. Stone patients without diabetes, as well as pregnant women, were excluded from this study. Patients were followed from their initial stone event until either recurrence of stone disease or until the end of the study period (April 2024), whichever occurred first.

Laboratory tests, blood and urine, were performed on all patients in this study before any urological procedure intended for lithiasis. Serum and urine analysis was conducted using an automatic biochemistry analyzer (Architect C4000 and Urisys 1800). BMI was calculated as weight (kg) divided by the square of height (m^2^).

The imaging assessment comprised ultrasonography, computed tomography, and, in specific situations, urography. The lithiasis fragments analyzed were samples collected following extracorporeal lithotripsy, percutaneous nephrolithotomy, spontaneous ureteric passes, or retrograde ureteroscopy.

The stones were analyzed in the Center for Morphological and Spectroscopic Analysis of Urinary Stones “Michel Daudon” in Iasi. The spectroscopic analysis was performed using the Bruker Alpha II FT-IR spectrometer (Bruker Corporation, Billerica, MA, USA) for stone composition analysis. Potassium bromide was used as a reagent to prepare stone samples for analysis.

A representative sample was crushed and mixed with potassium bromide for spectroscopic analysis. The spectra obtained were compared with reference spectra of stones with known compositions. Stones were classified according to Michel Daudon into calcium oxalate monohydrate, calcium oxalate dihydrate, calcium phosphate, pure uric acid, mixed uric acid and mixed stones [9]. 

The stones were categorized into different types based on their composition, including calcium oxalate (in mono- and dihydrate types), calcium phosphate, mixed uric acid (if the uric acid component accounted for more than 70% of the total stone), pure uric acid, and mixed stones, of which the majority comprised between 50 and 69% of the total.

All participants were informed about the objectives, methods, and benefits of the study. Written informed consent was obtained before any study-related procedure was conducted. The informed consent process complied with the principles outlined in the Declaration of Helsinki and was approved by the Ethics Committee of Dr. C. I. Parhon Hospital (approval number: 3350/6 May 2022).

Statistical analysis was conducted using SPSS software version 27 (IBM Corp., New York, NY, USA). Continuous variables were expressed as means ± standard deviations, while categorical variables were presented as frequencies and percentages. Group comparisons were made using the χ^2^ test for categorical variables, and in instances where sample sizes were small (less than five cases per group), Fisher’s exact test was applied.

A Kaplan–Meier analysis was used to evaluate the time to stone recurrence, and survival curves were compared using the log-rank test (Mantel–Cox). A significance level of *p* < 0.05 was set to determine statistical significance.

## 3. Results

Between April 2017 and April 2024, 532 patients with lithiasis and diabetes were treated in our clinic. Morphological and spectroscopic analysis was performed on 128 patients aged 19–79 (mean age 60.68 ± 11.96).

Most of the patients diagnosed with diabetes and urinary lithiasis were over 50 years old (82.04%). The prevalence of diabetes among young patients was low, with 5.46% occurring in those aged 19–30 and 31–40.

As Table 1 indicates, there were no significant differences in the environmental backgrounds of individuals with type 1 diabetes when compared to those with type 2 diabetes. The body mass index (BMI) was significantly higher in patients with type 1 diabetes compared to those with type 2 diabetes. A noteworthy correlation was found between a history of urine infections and both forms of diabetes, indicating that individuals with type 1 diabetes encountered a higher incidence of urinary infections.

As Table 2 shows, six categories of biochemical compositions of stones were identified, the most frequent being stones of mixed uric acid (MUA)—52.3%, pure uric acid (PUA)—10.9%, mixed stones (MS)—12.5%, calcium oxalate monohydrate (COM) and dihydrate (COD)—9.37% each, and carbapatite (CA)—5.46%.

Significant differences were found regarding body mass index, abdominal circumference, and personal history of urinary stones. A total of 62.7% of patients with MUA and 78.6% of those with PUA had a history of stones, compared to 25% of those with COM and 14.3% with CA. As Table 2 shows, the distribution of urinary stone compositions varies significantly between T1D and T2D patients. MUA stones are more commonly found in the T2D group, while PUA stones are more prevalent in the T1D group, the difference being statistically significant (*p* ≤ 0.001). Despite the fact that diabetic patients frequently present urinary infections associated with poor immune status, in our research, we did not identify stones with struvite composition in this group of patients.

Table 3 indicates that regarding the associated pathologies, higher percentages of CKD were observed in patients with acid uric stones (PUA: 57.1%, MUA: 38.8%) compared to patients with other types of urolithiasis (COM: 25%, CA: 14.3%), the differences being statistically significant (*p* = 0.03). By referring to the associated hypertensive pathology, the distribution percentages between the stone groups were similar, with no significant statistical differences (*p* = 0.11).

As can be observed in Table 4, patients with T1D had poorer control of glycaemic values than patients with T2D, a statistically significant difference. Important differences were also recorded regarding serum creatinine and body mass indexes.

We conducted a comparative analysis, considering the stones’ biochemical composition and the values of various biological parameters, and identified a series of significant elements (Table 5). Patients with uric acid stones had significantly higher mean creatinine values compared to the other types of stones (PUA: 1.51 mg/dL, MUA: 1.2 mg/dL compared to COM: 1.17 mg/dL and CA: 0.90 mg/dL, *p* < 0.001).

Concerning the lipid profile of the patient with diabetes and urolithiasis, there were no significant differences in HDL cholesterol levels according to stone composition. Patients with PUA and MUA urolithiasis had higher average triglyceride values (198.21 mg/dL and 168.68 mg/dL, respectively) compared to other types of stones (*p* ≤ 0.001). The mean urine pH found was within the pathological range for all types of stones, with the lowest values observed for patients with MUA and PUA (5.13 and 5.28, respectively). These differences were statistically significant compared to the other types of stones (*p* < 0.001).

The statistically significant difference in glycaemic control, as evidenced by average blood sugar levels (T1D: 133.21 mg/dL vs. T2D: 118.03 mg/dL, *p* ≤ 0.001), may influence the overall health outcomes of these patients. Furthermore, the duration of diabetes and the presence of other comorbidities, such as hypertension and chronic kidney disease, could also impact these differences. For instance, patients with T1D exhibited a higher mean serum creatinine level (1.34 mg/dL) compared to those with T2D (1.11 mg/dL, *p* = 0.001), which could reflect poorer kidney function associated with prolonged glycaemic exposure. Notably, patients with PUA stones, primarily from the T1D group (PUA: 57.1%), exhibited poorer glycaemic control, which is likely contributing to elevated uric acid levels and subsequent stone formation. Conversely, T2D patients with MUA stones showed a different clinical profile, characterized by a lower prevalence of chronic kidney disease (CKD) (MUA: 38.8%) compared to those with PUA stones. This suggests that the metabolic dysregulation associated with T1D may predispose patients to specific types of urolithiasis.

Table 6 indicates that seven types of antidiabetic medications were identified among this patient group. In our study, the antidiabetic medication utilized for the management of diabetes does not appear to influence the biochemical composition of the stones as determined by spectroscopic analysis. Specifically, for patients with T1D, there were no instances of oral medications such as Metformin, Pioglitazone, or Sulfonylureas; the predominant treatment involved insulin, with a total of 24 patients on insulin therapy. In contrast, patients with T2D exhibited a broader range of medication usage, with 27 patients treated with various combinations, including Metformin and Sulfonylureas. Despite the differing treatment regimens, statistical analysis revealed no significant association between the type of medication and the biochemical composition of the stones (*p* = 0.58), indicating that the prescribed antidiabetic treatments do not correlate with the specific types of kidney stones formed in these patients.

**Figure 1:**
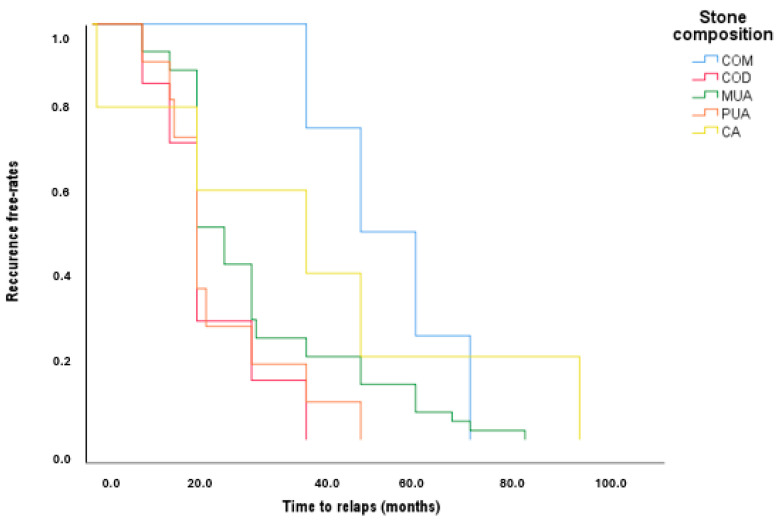
Kaplan–Meyer: the first manifestation of the lithiasis disease to relapse. *p* = 0.034.

In the Kaplan–Mayer curve from Figure 1, we analyzed the recurrence times of urinary lithiasis by referring to the composition of the stones determined spectroscopically. The Kaplan–Meier analysis reveals that uric acid stones, both MUA and PUA, significantly increase the recurrence risk in diabetic patients. Patients with PUA stones have a median time to recurrence of just 24 months, with a rapid decline in recurrence-free survival, indicating an early and frequent relapse. Similarly, those with MUA stones show a median time of 30 months, with a recurrence rate within the first 40 months.

In contrast, COM stones, with a median time to relapse of 60 months, demonstrate a far lower recurrence rate. Thus, identifying the composition of uric acid stones early in diabetic patients is crucial to effectively managing and reducing the high likelihood of recurrence.

## 4. Discussion

Our study highlighted the relationship between diabetes and renal stone composition. The spectroscopic analysis identified mixed uric acid stones as the most prevalent, particularly among T2D patients, while pure uric acid stones were more frequently observed in T1D patients. Notably, patients with PUA stones demonstrated poorer glycaemic control, reflected in higher average blood sugar levels and serum creatinine, compared to T2D patients. Additionally, the study found significant differences in the recurrence patterns of urinary lithiasis based on stone composition, suggesting that metabolic dysregulation in diabetes may predispose individuals to specific types of stones and influence recurrence risks

Urinary lithiasis is a highly prevalent disease that affects an important percentage of people worldwide, with an incidence rate ranging from 5% to 10%. There has been a significant rise in the prevalence of urinary lithiasis among the general population. This rise can be attributed to multiple environmental variables, such as reduced fluid consumption and diets rich in salt and proteins [1,10].

Recent research indicates that, alongside the conventional risk factors associated with the occurrence of urinary stones, metabolic syndrome plays a significant part in both the development and recurrence of this illness. Each component of the metabolic syndrome, including abnormal blood sugar levels, obesity, high blood triglyceride levels, and insulin resistance, is considered an independent risk factor [10,11]. The incidence of the metabolic syndrome may vary depending on features such as age, gender, and ethnicity. It impacts a significant number of adults, ranging from 24% to 42%, and is considerably more prevalent among elderly individuals, with over 66.4% affected [12].

Several studies have documented a correlation between diabetes and the formation of kidney stones. In an important cross-sectional study, Taylor et al. emphasize the connection between diabetes and the occurrence and recurrence of urinary stones. In addition, the authors noticed a link between the detection of a urinary stone and an increased likelihood of being diagnosed with diabetes [13]. Daudon et al. investigated the way diabetes influences the composition of urinary stones. Their results showed that diabetes is an important risk factor for developing uric acid lithiasis. Additionally, the study indicated that uric acid lithiasis is particularly common among individuals with type 2 diabetes, mainly when associated with additional characteristics of the metabolic syndrome. Also, Daudon suggests that type 2 diabetes was three times more common in patients with uric acid lithiasis than in patients in whom the spectroscopic analysis of the stones revealed calcium oxalate lithiasis [7]. In another study, Pak et al. indicate that 33.9% of patients with urinary stones and diabetes have uric acid stones, while in diabetic patients, the incidence of this biochemical type of stone was only 8.5% [14]. The data acquired in this research are consistent with the findings reported in the literature. In this group of patients with both lithiasis and diabetes, the spectroscopic analysis of the stones revealed that uric lithiasis was the most prevalent type, accounting for 63.27% of cases. The other biochemical species of calculi were present in significantly lower proportions. The high occurrence of uric acid stones in diabetics can be attributed to insulin resistance, a characteristic feature of diabetes. Insulin is known for its property to stimulate the synthesis of ammonia and the sodium-hydrogen exchange in the renal tubule, which aids in the excretion of ammonium in urine. Diabetic patients commonly have insulin resistance, resulting in reduced ammonia production and reduced urinary pH [13,14]. Furthermore, studies have demonstrated that insulin can increase uric acid and sodium reabsorption in the proximal convoluted tubule, resulting in higher uric acid levels in the blood and reduced excretion of uric acid and sodium [15,16]. Regarding an additional important feature of the metabolic syndrome, specifically obesity, our study revealed that most patients were overweight. Moreover, in patients with uric acid lithiasis (MUA and PUA stones), the mean weight was significantly higher than that of the other patient groups. A significant percentage of patients diagnosed with diabetes are either obese or overweight. The presence of excessive secondary adipose tissue leads to an increase in insulin resistance due to an excess of pro-inflammatory cytokines. Therefore, the additional weight in these patients leads to an exacerbation of ammonia genesis and, thus, a reduction in urine pH [17].

Analyzing the urinary pH of the patients in our study, we observed that the pH values were abnormal for all biochemical subtypes of stones, indicating the presence of acidic urine. However, patients with spectroscopic analysis indicating MUA or PUA stones had pH values significantly below the group’s average. Reichard et al. published a study analyzing the characteristics of patients with pure uric acid lithiasis. They emphasize that this category of patients is characterized by higher body mass indices, increased serum uric acid levels, and slightly higher urinary pH [18]. Regarding our study, patients with PUA-type stones had greater BMI and abdominal circumferences than the general group. However, these patients had slightly elevated values regarding urinary pH compared to patients with MUA-type stones. In another investigation, Friedlander et al. investigated the metabolic profiles of patients with different types of urinary stones. They analyzed a group of 232 patients and found that 35.8% of them had pure uric acid stones. Similar to the previous research, these patients had lower urinary pH and higher BMI than the control groups [19].

Regarding the lipid profile analysis of diabetic and lithiasis patients in our group, we did not observe any features that reached the threshold of statistical significance when compared to the average HDL cholesterol values among the different stone compositions. Considering the mean blood triglyceride levels, our data show that patients with MUA and PUA-type stones had higher values than the group average. In an important epidemiological analysis published by Qin et al. regarding the implications of the lipid profile in the etiopathogenesis of urinary lithiasis, the authors state that a higher triglyceride–glucose index was independently associated with an increased likelihood of nephrolithiasis and nephrolithiasis recurrence [20]. In a cross-sectional study, Chen et al. investigated the lipid profile of patients with metabolic syndrome, lithiasis, and non-lithiasis, emphasizing biological variables. It was observed that the patients without lithiasis exhibited higher levels of HDL cholesterol and triglyceride compared to the patients with lithiasis. Also, the study found that patients with lithiasis had significantly higher blood triglyceride levels than those without lithiasis [11].

We analyzed the chronic medication prescribed to diabetic patients. The majority of patients diagnosed with type 2 diabetes were prescribed Metformin either as an independent treatment or in combination with other drugs (Pioglitazone, Sulfonylureas or Dapagliflozin). Our analysis did not find any significant statistical differences in the correlation between therapeutic schemes and the composition of the stones. The available data on the relationship between oral antidiabetics and the development of kidney stones are rather conflicting. According to a recent study by Yang et al., Metformin has been found to have multiple benefits in urinary biochemistry. These effects include reducing crystal sedimentation, decreasing the expression and production of inflammatory genes OPN (osteopontin) and MCP-1 (monocyte chemoattractant protein 1), reducing renal tubular injury and crystal deposition, suppressing inflammatory responses, decreasing urinary oxalate excretion, acting as an antioxidant, and reducing cytotoxicity [21]. Another recent study examining the effects of Biguanide administration on urinary parameters found that using Metformin by diabetic patients and kidney stones does not seem to cause any significant changes in these urinary parameters. However, the use of Metformin in combination with other diabetic medications was found to be associated with higher levels of urinary citrate [22]. However, another study examining the influence of Metformin on urinary parameters concluded that Metformin had an acidifying effect on urine, which may have a negative impact on uric acid nephrolithiasis, the most common form of kidney stone in individuals with type 2 diabetes [23].

As far as we know, no comparative analyses have been carried out on the urinary stones of patients with T1D versus T2D. The data regarding the crystalluria of patients with T2D highlight the fact that the most frequent crystals found in the urine of this category of patients are represented by calcium oxalate dihydrate, uric acid, and tyrosine [24]. However, the importance of this information should be considered limited as it only provides information on the composition of the external layers of the stones; therefore, it is an unreliable method of determining the biochemical composition of the stones.

A meta-analysis published by Geraghty et al. shows that, in general, the risk of urinary lithiasis in diabetic patients is due, on the one hand, to chronic hyperglycaemia, specific to both types of diabetes, and, on the other hand, to the glucose tolerance in type 2 diabetes [25]. In our research, a fourth of the patients had type 1 diabetes, and compared to type 2 diabetic patients, they were characterized by a poorer control of glycaemic values. Also, patients with type 1 diabetes were diagnosed earlier compared to patients with type 2 diabetes (46.2 versus 52.5 years). Our previous study showed that diabetic patients with urinary lithiasis, who also have hypertension and excess weight, are 4.3 times more likely to experience a recurrence of lithiasis compared to those without the combined set of diseases [9]. Our present research examined the period between the initial occurrence of stone disease and subsequent relapse. As indicated by spectroscopic analysis, we noticed that patients with uric acid stones encountered shorter intervals between relapses than patients with other types of stone biochemistry. A major investigation conducted by Daudon and colleagues examines how the biochemical composition of stones affects the probability of lithiasis recurrence. The study reveals that cystine and dihydroxyadenine stones, which are caused by genetic disorders that lead to significant changes in urinary biochemistry, have a high rate of relapse. On the other hand, uric acid stones result from intense metabolic processes and have a high recurrence rate (over 50%), in contrast to 30% for COM stones and 41% for COD stones [26]. Uric acid lithiasis has an increased probability of recurrence due to its significant metabolic activity. In cases where patients frequently suffer from diabetes, hyperuricemia, hypertension, obesity, and dyslipidaemia, they frequently suffer from various degrees of kidney injury. Once there is also a possible obstruction due to kidney stones, it can have negative effects on kidney function, potentially leading to irreversible consequences [9,27]. In addition to the increased risk of lithiasis recurrence, the lithiasis and diabetic patient can raise significant treatment problems, because extracorporeal lithotripsy often requires the injection of contrast during the procedure, fragmentation is problematic in the context of frequently associated obesity, and the patient’s adherence to prescribed metaphylactic measures is often reduced in the context of polymedication [28,29,30].

Our research is limited by its dependence on retrospective data collection methods, which may include biases that result from incomplete records or mistakes in self-reported histories. Another possible limitation of our research may be the lack of information provided by the 24 h urine analysis (urinary citrate and oxalate values), but from the point of view of the etiological diagnosis of stone, the spectroscopic analysis can provide relevant information.

## 5. Conclusions

Diabetes mellitus is a health issue that is becoming more common, and it is a significant risk factor for the development and recurrence of urinary stones. Our data suggest that patients with type 2 diabetes have a higher prevalence of mixed uric acid stone. In contrast, type 1 diabetes patients predominantly form pure uric acid stones, which significantly impacts the dissolution strategy adopted in the case of pure stones. Furthermore, it is essential to properly inform patients about the significance of maintaining appropriate urine pH and glucose levels to reduce the likelihood of lithiasis recurrence.

## Figures and Tables

**Table 1:** Demographic characteristics.

Characteristics	Whole Sample (n = 128)	Type 1 Diabetes (n = 32)	Type 2 Diabetes (n = 96)	*p*
Age (mean ± SD)	60.68 (±11.96)	63.56 (±10.04)	59.71 (±12.43)	0.58
Gender	Male—79 (61.7)	Male—18 (14.1)	Male—61 (47.7)	0.68
	Female—49 (38.3)	Female—14 (10.9)	Female—35 (27.3)	
Environment	Urban (%)—61 (47.7)	13 (10.2)	48 (37.5)	0.35
	Rural (%)—67 (52.3)	19 (14.8)	48 (37.5)	
BMI kg/m^2^ (mean ± SD)	28.30 (±4.64)	30.45 (±5.87)	27.50 (±3.87)	0.02
Abdominal circumference cm (SD)	103.72 (±11.68)	102.92 (±11.95)	101.83 (±7.41)	0.10
Family history of urinary stones	No—108 (84.4%)	24 (18.8)	84 (65.6)	0.9
	Yes—20 (15.6)	8 (6.3)	12 (9.4)	
History of urinary stones	No—57 (44.5)	18 (14.1)	39 (30.5)	0.26
	Yes—71 (55.5)	39 (30.5)	57 (44.5)	
History of urinary infection	No—79 (61.7)	15 (11.7)	64 (50.0)	0.04
	Yes—49 (38.3)	17 (13.3)	32 (25.0)	

**Table 2:** Spectroscopic analysis of urinary stones in diabetic patients.

Stone Composition	T1D		T2D		Total		
	n	%	n	%	n	%	
COM	3	9.4	9	9.4	12	9.4	*p* ≤ 0.001
COD	1	3.1	11	11.4	12	9.4	
MUA	8	25	56	58.3	67	52.3	
PUA	14	43.7	3	3.12	14	10.9	
CA	2	6.2	5	5.2	7	5.5	
MS	4	12.5	12	12.5	16	12.5	
TOTAL	32	25	96	75	128	100	

**Table 3:** Chronic pathologies.

Characteristics	Whole Sample (n = 128)	COM (n = 12)	COD (n = 12)	MUA (n = 67)	PUA (n = 14)	CA (n = 7)	MS (n = 16)	*p*
Diabetes	T1D (%)—32 (25)	3 (25)	1 (8.3)	11 (16.4)	11 (78.6)	2 (28.6)	4 (25)	<0.001
	T2D (%)—96 (75)	9 (75)	11 (91.7)	56 (83.6)	3 (21.4)	5 (71.4)	12 (75)	
Hypertension	No (%)—77 (60.2)	7 (58.3)	9 (75)	43 (64.2)	10 (71.4)	3 (42.9)	5 (31.3)	0.11
	Yes (%)—51 (39.8)	5 (41.7)	3 (25)	24 (35.8)	4 (28.6)	4 (57.1)	11 (68.8)	
Chronic Kidney Disease	No (%)—85 (66.4)	9 (75)	12 (100)	41 (61.2)	6 (42.9)	6 (85.7)	11 (68.8)	0.03
	Yes (%)—43 (33.6)	3 (25)	-	26 (38.8)	8 (57.1)	1 (14.3)	5 (31.3)	

**Table 4:** Comparative analysis of clinical and biological characteristics in patients with type 1 diabetes vs. type 2 diabetes.

Characteristics	Mean Age (SD)	Age at the Time of Diabetes Diagnosis (Years)	BMI (kg/m^2^)	Urinary pH	Average Blood Sugar (mg/dL)	Triglycerides (mg/dL)	HDL (mg/dL)	Serum Creatinine (mg/dL)
T1D	63.56 (10.04)	46.22 (9.70)	30.45 (5.87)	5.45 (0.54)	133.21 (16.31)	170.28 (35.93)	87.65 (10.55)	1.34 (0.38)
T2D	59.72 (12.43)	52.52 (12.42)	27.58 (3.92)	5.40 (0.49)	118.03 (16.36)	160.54 (27.95)	87.43 (6.95)	1.11 (0.31)
*p*	0.11	0.01	0.002	0.65	≤0.001	0.11	0.89	0.001

**Table 5:** Serum and urine parameters in lithiasic and diabetic patient.

		Mean (SD)	95% CI		*p*
Creatinine (mg/dL)	COM (n = 12)	1.17 (0.30)	0.9794	1.3706	<0.001
	COD (n = 12)	0.87 (0.16)	0.7747	0.9836	
	MUA (n = 67)	1.20 (0.33)	1.1232	1.2857	
	PUA (n = 14)	1.51 (0.27)	1.3624	1.6747	
	CA (n = 7)	0.90 (0.33)	0.5933	1.2067	
	MS (n = 16)	1.05 (0.34)	0.8684	1.2353	
	TOTAL (n = 128)	1.16 (0.35)	1.1086	1.2311	
Urinary pH	COM (n = 12)	5.66 (0.53)	5.3258	6.0076	<0.001
	COD (n = 12)	5.62 (0.43)	5.3499	5.9001	
	MUA (n = 67)	5.28 (0.46)	5.1791	5.3881	
	PUA (n = 14)	5.13 (0.73)	5.0150	5.5564	
	CA (n = 7)	5.42 (0.13)	4.7517	6.1055	
	MS (n = 16)	5.75 (0.54)	5.4581	6.0419	
	TOTAL (n = 128)	5.41 (0.50)	5.3296	5.5063	
Average blood sugar (mg/dL)	COM (n = 12)	119.16 (15.26)	109.4669	128.8664	0.05
	COD (n = 12)	122.33 (20.36)	109.3960	135.2707	
	MUA (n = 67)	120.74 (14.08)	117.3110	124.1815	
	PUA (n = 14)	135.14 (20.96)	123.0403	147.2454	
	CA (n = 7)	120.71 (26.57)	96.1364	145.2922	
	MS (n = 16)	116.81 (20.01)	106.1460	127.4790	
	TOTAL (n = 128)	121.82 (17.57)	118.7539	124.9023	
Triglycerides (mg/dL)	COM (n = 12)	140.08 (20.49)	127.0637	153.1030	<0.001
	COD (n = 12)	138.91 (21.01)	125.5658	152.2676	
	MUA (n = 67)	168.68 (27.08)	162.0797	175.2935	
	PUA (n = 14)	198.21 (25.89)	183.2602	213.1684	
	CA (n = 7)	148.71 (26.34)	124.3461	173.0825	
	MS (n = 16)	149.68 (24.08)	136.8519	162.5231	
	TOTAL (n = 128)	162.97 (30.29)	157.6782	168.2749	
HDL (mg/dL)	COM (n = 12)	88.83 (14.38)	79.6946	97.9720	0.35
	COD (n = 12)	87.08 (8.38)	81.7574	92.4093	
	MUA (n = 67)	88.64 (7.29)	86.8631	90.4204	
	PUA (n = 14)	83.92 (6.79)	80.0030	87.8541	
	CA (n = 7)	85.57 (4.85)	81.0767	90.0661	
	MS (n = 16)	85.93 (5.11)	83.2102	88.6648	
	TOTAL (n = 128)	87.49 (7.96)	86.0999	88.8845	

**Table 6:** Antidiabetic treatment followed by the patient.

Diabetes Type	Stone Type	MTF	PIO	SLF	MTF + SLF	MTF + DPG	INS	INS + MTF	λ^2^	*p*
T1D	COM	0	0	0	0	0	3	0	22.67	0.01
	COD	0	0	0	0	0	1	0		
	MUA	0	0	0	0	0	14	0		
	PUA	0	0	0	0	0	1	5		
	CA	0	0	0	0	0	2	0		
	MS	0	0	0	0	0	3	3		
	TOTAL	0	0	0	0	0	24	8		
T2D	COM	2	1	2	4	2	1	0	22.94	0.58
	COD	19	0	6	16	14	0	1		
	MUA	0	3	0	3	0	0	0		
	PUA	2	0	1	2	0	0	0		
	CA	4	0	2	5	1	0	0		
	MS	0	2	0	0	0	3	0		
	TOTAL	27		11	30	17	4	1		

MTF—Metformin, PIO—Pioglitazone, SLF—Sulfonylureas, DPG—Dapagliflozin, INS—insulins.

## Data Availability

The data that support the findings of this study are available from the corresponding author upon reasonable request.
